# A real-world approach to Evidence-Based Medicine in general practice: a competency framework derived from a systematic review and Delphi process

**DOI:** 10.1186/s12909-017-0916-1

**Published:** 2017-05-03

**Authors:** Kevin Galbraith, Alison Ward, Carl Heneghan

**Affiliations:** Centre for Evidence-based Medicine, Nuffield Department of Primary Care Health Sciences, University of Oxford, Radcliffe Primary Care Building, Radcliffe Observatory Quarter, Oxford, OX2 6GG UK

**Keywords:** General practice, Evidence-based medicine, Systematic review, Delphi process, Competence

## Abstract

**Background:**

Evidence-Based Medicine (EBM) skills have been included in general practice curricula and competency frameworks. However, GPs experience numerous barriers to developing and maintaining EBM skills, and some GPs feel the EBM movement misunderstands, and threatens their traditional role. We therefore need a new approach that acknowledges the constraints encountered in real-world general practice. The aim of this study was to synthesise from empirical research a real-world EBM competency framework for general practice, which could be applied in training, in the individual pursuit of continuing professional development, and in routine care. We sought to integrate evidence from the literature with evidence derived from the opinions of experts in the fields of general practice and EBM.

**Methods:**

We synthesised two sets of themes describing the meaning of EBM in general practice. One set of themes was derived from a mixed-methods systematic review of the literature; the other set was derived from the further development of those themes using a Delphi process among a panel of EBM and general practice experts. From these two sets of themes we constructed a real-world EBM competency framework for general practice.

**Results:**

A simple competency framework was constructed, that acknowledges the constraints of real-world general practice: (1) mindfulness - in one’s approach towards EBM itself, and to the influences on decision-making; (2) pragmatism – in one’s approach to finding and evaluating evidence; and (3) knowledge of the patient – as the most useful resource in effective communication of evidence. We present a clinical scenario to illustrate how a GP might demonstrate these competencies in their routine daily work.

**Conclusion:**

We have proposed a real-world EBM competency framework for general practice, derived from empirical research, which acknowledges the constraints encountered in modern general practice. Further validation of these competencies is required, both as an educational resource and as a strategy for actual practice.

**Electronic supplementary material:**

The online version of this article (doi:10.1186/s12909-017-0916-1) contains supplementary material, which is available to authorized users.

## Background

Evidence-Based Medicine (EBM) has become established as a prominent paradigm in the field of healthcare, [1] and is defined as ‘the conscientious, judicious and explicit use of current best evidence in making decisions about the care of individual patients’ [2]. In 1992, a five-step model for EBM was first proposed, [1] and in 2005 a core curriculum for the teaching and practice of EBM was outlined [3]:

1. Translation of uncertainty to an answerable question
2. Systematic retrieval of best evidence available
3. Critical appraisal of evidence for validity, clinical relevance, and applicability
4. Application of results in practice
5. Evaluation of performance

These five sequential steps are included in several general practice curricula, such as those published by the Royal College of General Practitioners (RCGP) in the UK, [4] and the Royal Australian College of General Practitioners (RACGP); [5] and most are included in the competency frameworks published by the Accreditation Council for Graduate Medical Education for Family Medicine in the U.S., [6] and the College of Family Physicians of Canada [7]. However, there is a body of opinion amongst GPs that these steps fail to take into account the complexities of primary care [8]. As an example, several surveys exploring GPs’ views on the most appropriate EBM strategy in general practice reported that the use of pre-appraised secondary sources of evidence was preferred over searching (step 2) and appraising evidence themselves (step 3) [9–11]. GPs have also described a number of barriers to use of evidence, such as time constraints, [9–15] patients’ expectations [9–14] and insufficient evidence relevant to general practice [9–11, 13, 14, 16]. None of the competency frameworks or curricula mentioned here make any explicit acknowledgement of these time and workload barriers. To do so might be inappropriate given their application in clinical governance. However, GPs might arguably become more engaged with evidence if future development of competency frameworks are more relevant to practice, and acknowledge the constraints encountered in the real world. The aim of this study was therefore to generate a simple and elegant competency framework that could guide EBM learning in general practice, and equip individual GPs with a feasible approach to EBM in their routine work.

## Methods

We identified empirical research, published in English between 1991 (when EBM first appeared in the literature [17]) and August 2011, reporting on general or family practitioners, and on competence relating to EBM as an overarching approach to patient care. Beyond the scope of this review were studies that addressed specific elements of EBM or a specific clinical topic, and studies evaluating educational interventions. We searched the following databases: MEDLINE, Embase, Cochrane Central Register of Controlled Trials, ERIC, Australian Education Index, British Education Index, HMIC, PsychINFO, Science Citation Index and Social Science Citation Index. Search terms comprised ‘general practitioner,’ ‘evidence-based practice,’ ‘competence’ and all the variations and synonyms of theses terms that could be gleaned from the Oxford Dictionary & Thesaurus, [18] and from an initial scoping of known key studies in this area. Relevant articles were selected initially by examination of the title and abstract, and then by scrutiny of their full text. The reference list of each included article was hand-searched for additional relevant articles. The quality of included studies was assessed using a combination of frameworks as described by van Dijk et al.: [19] for quantitative studies, a modified version of the Strengthening the Reporting of Observational Studies in Epidemiology (STROBE) statement [20] was used, and for qualitative studies, a modification of criteria proposed by Giacomini and Cook [21, 22]. Studies were categorised as high/moderate/low quality according to the number of criteria fulfilled. As a result of resource limitations, only one reviewer made decisions on the selection of relevant studies, assessment of quality and extraction of data. We did not include data pertaining to GPs that were not distinct from other professional groups. Following the method of Pedersen et al., [23] we organised quantitative outcome data under descriptive headings, to facilitate later synthesis with qualitative data. We extracted qualitative data from text labelled ‘results’ or ‘findings.’ One reviewer synthesised qualitative data using ‘thematic synthesis,’ [24] and coded data using QRS NVivo, version 10. Codes with similar meanings were grouped into descriptive themes. Having described the data, we progressed to an analytic phase: for each descriptive theme, the question was posed: ‘How does this address the research question: ‘*what themes underpin the meaning of EBM competence in general practice?’* This resulted in a series of statements such as ‘GPs are reluctant to challenge expert opinion.’ The statements were themselves organised into a set of overarching ‘analytic’ themes. We organised quantitative survey outcome data under descriptive headings. Outcomes were synthesised with qualitative data if they were derived from surveys with low or moderate risk of bias, and either consistent across several surveys, or from a single survey with external validity. Each analytic theme derived from the qualitative synthesis was matched with quantitative survey outcome data under relevant descriptive headings. This allowed triangulation of analytic themes with survey data, and provided additional useful insights to several of the analytic themes. We carried out sensitivity analyses to test the effect of including and excluding studies of poorer quality, using to the method described by Thomas and Harden: first we reviewed the quality of studies contributing data to each of the analytic themes derived from the qualitative synthesis; then for each analytic theme, a decision was made regarding the extent to which it depended upon data from less robust studies [24]. Themes were mapped to the core principles of adult learning theory [25] to generate a preliminary competency framework, which could serve as a ‘straw model’ for experts to work on in the next stage of the study. A two-round Delphi process was conducted among a panel of ten experts in general practice and EBM (one of whom was the primary investigator). Half of the panelists were experienced full time general practitioners, and half were academic clinicians with experience in general practice, medical education and relevant EBM research. A decision committee convened after each round to collate findings. The committee comprised the principal investigator, and three members of the academic staff of the Department of Primary Care Health Sciences at the University of Oxford, one of whom was a member of the expert panel. In Round 1, the preliminary competency framework (‘straw model’) was rated by panelists for importance, achievability and demonstrability. In Round 2, a revised framework was rated, and component competencies were ranked across the same dimensions of importance, achievability and demonstrability. Concordance of rankings between panelists was estimated by Kendall’s W (coefficient of concordance). Free-text comments were invited in each round. Changes in mean overall score and standard deviation were calculated for each competency, between Round 1 and Round 2. A consensus framework was generated in light of the data from both rounds. This, and the free-text comments were treated as qualitative data and analysed thematically by one reviewer, using the methods described for the systematic review. The credibility of emergent themes was checked against quantitative findings. Finally, these emergent themes were synthesised with the themes derived from the initial mixed-methods systematic review. This generated a final framework of three competencies for use in GP training and continuing professional development, and that could be used as an approach to EBM in routine practice.

## Results

**Fig. 1:**
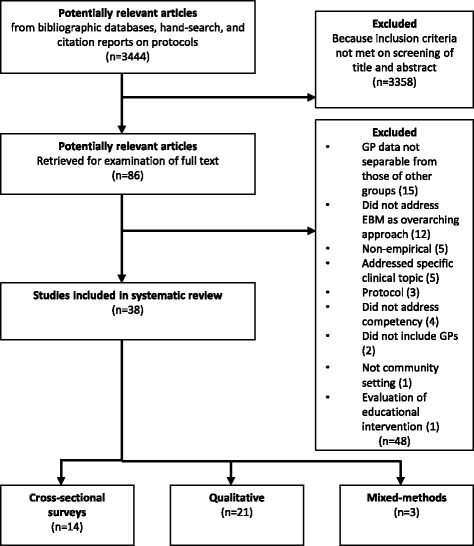
Flowchart for selection of studies

The systematic review yielded 3444 articles, of which 38 were considered suitable for inclusion (Fig. 1). These comprised 14 quantitative studies (all cross-sectional surveys); 21 qualitative studies; and three studies of mixed methods (Additional file 1). Among the qualitative methods, the most commonly applied were interviews (14 studies) and focus groups (eight studies); only one study used direct observation of GPs at work. Publication dates ranged between 1995 and 2009. Most of the studies (22) were conducted in UK. Five were conducted in Australia, three in Canada, two in US and one in Jordan, Turkey, Spain,

Belgium, Sweden and Germany. The 38 included studies comprised a combined sum of over 3500 GP participants (Table 1). The most widely reported characteristic was sex distribution, with 65% male and 35% female across 25 studies reporting these data specifically for GPs. The mean age across only eight studies that included specific GP data was 44.4 years (95% CI 42.3 to 46.4). The proportion aged below 40 was 37% (combined data from only 6 studies). 33% worked part-time (combined data from nine studies); and 73% worked in group practices (combined data from 11 studies).

**Table 1:** Characteristics of GP subjects

Variable(s) reported		^a^No. studies reporting specific GP data	Summary statistic combining specific GP data
Age	Mean	8	44.4 (95% CI 42.3, 46.4)
	No. (%) aged <40	6	357/965 (37%)
	No. (%) aged 40+		608/965 (63%)
Sex	No. (%) male	25	1274/1946 (65%)
	No. (%) female		672/1946 (35%)
Work pattern	No. (%) full-time	9	466/699 (67%)
	No. (%) part-time		233/699 (33%)
Personnel	No. (%) working solo	11	194/826 (23%)
	No. (%) working in group practice		632/826 (77%)
GP training activity in practice	No. (%) working in training practice	3	206/664 (31%)
Experience	Mean years in practice	6	16 (95% CI 15, 17)
Postgraduate GP qualification	No. (%) holding postgraduate GP qualification	7	776/1272 (61%)
Prior EBP training	No. (%) trained in searching literature	3	90/573 (16%)
	No. (%) trained in critical appraisal	4	254/810 (31%)
	No. (%) trained in unspecified EBP skills	2	141/248 (57%)

^a^“Specific GP data” refers to data that are separable from those of other professional groups

### Quality of included studies

Most of the studies (*N* = 31) were considered to be of moderate quality (Table 2). Four studies met all the quality criteria and were therefore regarded as high quality. Two were surveys (one in UK, [10] and the other in Jordan [13]), using the same validated, self-administered questionnaire to investigate EBM knowledge, skills, attitudes and behaviour among GPs. The other two were qualitative studies: a focus group conducted among Belgian GPs, which investigated barriers to EBM; [26] and a Canadian interview study investigating EBM attitudes and experience, and decision making [27]. Three studies met fewer than half the criteria and were considered to be of low quality. Among the surveys, the most common methodological problems were a failure to explore reasons for non-participation and losses to follow-up, and an inadequate description of the design and quality of the survey instrument. Among the qualitative studies, the most common problems were found in analysis of data, specifically: a failure to iterate data collection to the point at which no further new findings arose (‘saturation’); and a failure to consolidate the credibility of findings by triangulating several different methods of data collection.

**Table 2:** Quality of included studies (*n* = 38)

	Quantitative methods							Qualitative methods					
Author/year	Specific objective/ hypothesis	Appropriate eligibility/ selection criteria	Important outcomes included	Instrument design/quality described	Response rate	Exploration of failure to participate	Clear presentation of results	Clear objective	Selection of participants	Methods to generate data	Data collection	Analysis of data	Clear presentation of results
Barghouti F, 2009 [13]	+	+	+	+	+	+	+						
Callen J, 2006 [9]	+	+	+	+	−	−	+						
Hannan A, 1998 [69]	+	−	+	−	−	−	+						
Kahveci R, 2009 [44]	+	+	+	+	−	−	+						
McColl A, 1998 [10]	+	+	+	+	+	+	+						
McKenna H, 2004 [16]	+	+	+	+	+	−	+						
Robinson G, 2000 [70]	+	+	+	−	+	−	+						
Salisbury S, 1998 [71]	+	+	−	−	+	+	+						
Samuel O, 1997 [50]	−	+	−	−	−	−	−						
Siriwardena A, 2007 [72]	+	+	+	+	−	−	+						
Taylor J, 2002 [14]	+	+	+	−	+	−	−						
Tracy C, 2003 [73]	+	+	+	+	−	+	+						
Trevena L, 2007 [12]	+	+	+	−	+	−	−						
Upton D, 2006 [15]	+	+	+	−	+	−	+						
Adams J, 2000 [29]								+	+	+	−	−	+
Armstrong D, 2002 [45]								−	+	+	−	−	+
Calderón C, 2011 [28]								+	±	+	+	−	+
Ely J, 2002 [30]								+	+	+	+	±	+
Ford S, 2002 [31]								+	−	+	+	±	+
Ford S, 2003 [42]								+	+	+	±	−	+
Freeman A, 2001 [32]								+	+	+	±	−	+
Gabbay J, 2004 [46]								+	+	+	+	±	+
Hall L, 1999 [33]								+	+	+	±	−	+
Hannes K, 2005 [26]								+	+	+	+	+	+
Lipman T, 2004 [74]								+	+	+	+	±	+
Lorenz K, 2005 [47]								+	+	+	±	−	+
Mayer J, 1999 [35]								+	+	+	+	±	+
Mears R, 2000 [8]								+	+	±	−	−	+
Putnam W, 2002 [37]								+	+	+	+	±	+
Short D, 2003 [39]								+	+	+	±	−	+
Skoglund I, 2007 [40]								−	+	±	±	−	+
Summerskill W, 2002 [41]								+	+	+	+	−	+
Tomlin Z, 1999 [75]								+	+	+	+	−	+
Tracy C, 2003 [27]								+	+	+	+	+	+
Wood F, 1995 [49]								+	+	+	+	−	−
Patterson J, 1999 [36]	+	+	+	+	+	−	+	+	+	+	±	−	+
Rohrbacher R, 2009 [38]	+	+	+	−	−	−	−	−	+	−	−	−	−
Young J, 2001 [11]	+	+	+	+	+	n/a	+	+	+	+	−	−	−

Opinion of the reviewer regarding quality: + fulfilled criterion; ± equivocal; − did not fulfill criterion; n/a not applicable as response rate 100%

### Themes derived from the mixed-methods systematic review

From the synthesis of qualitative studies, we identified seven literature-derived themes that address EBM competence in a general practitioner: (1) attitude towards EBM; (2) major influences on decision making; (3) use of pre-appraised sources of evidence; (4) integration of evidence and clinical expertise; (5) communication skills; (6) skill in searching for primary sources of best evidence; and (7) skill in critical appraisal of evidence. The findings relating to each theme are described in more detail below.

### Attitude towards EBM

There was a widespread perception that EBM fails to acknowledge the complexity and constraints of general practice: *‘One sees it at times as dogma... what I mean is that not all patients are the same and many times you are in front of a patient that EBM says has this or that, and you don’t see it’* [28]. This was evident from 19 qualitative studies [8, 11, 26–42]. Four surveys indicated that a minority of GPs (range 11% to 42%) perceived EBM to be of limited value in primary care, [9, 13, 14, 43] although these and four additional surveys found a clear majority of respondents felt positive or welcoming towards EBM [9–11, 13, 14, 36, 43, 44]. In a survey among 137 Australian GPs, 37 (27%) agreed that EBM could be described as ‘cookbook medicine,’ suggesting some perceived EBM to be rigid in its application [9]. Time constraint was widely perceived to be a barrier to EBM, [9–15] as were patients’ expectations [9–14] and a lack of relevance of research evidence to general practice [9–11, 13, 14, 16]. In one Australian survey of 107 GPs, 10 (9%) cited difficulty in tailoring evidence to the individual patient or general practice context [12]. Some GPs view EBM as a threat to their traditional role [26–28, 31, 34, 35, 38, 45]. However, there were no survey findings relating specifically to this opinion. Some GPs perceived that implementation of EBM is affected by a narrowing of their role, as specialists tighten evidential criteria for referral: *‘The last month I got two patients back... look, your patient does not fulfil our criteria and so he does not have to come. And I think: ‘Is that the kind of medicine I will be forced to do? That person comes with his complaints, whether he is fulfilling my criteria or not. But that will be the future task of the GP: helping the people who do not fit the criteria of the specialists’* [26]. GPs also associated EBM with restriction on individualised care, [29] restriction of therapeutic choices, [26–29, 38] and with devaluation of the ‘art’ (as opposed to the ‘science’) of medicine [27]. Some GPs viewed EBM as a form of reflective practice. One focus group found that GPs valued being encouraged to reflect on their decisions, prescribing habits, and perceptions of therapeutic freedom [26]. Another reported that EBM was regarded by some GPs as a means of critically reviewing personal clinical practice, and an opportunity to pose questions to be addressed using evidence from published research [28]. There were no surveys addressing this issue specifically.

### Major influences on decision-making

The doctor-patient relationship exerts a powerful influence on decisions. The desire to preserve it can outweigh considerations of evidence [8, 41]. In one survey of 60 Australian GPs, 44 (73%) agreed that ‘regardless of research findings, patients have unrealistic expectations which drive my treatment choices’ and 49 (82%) agreed that ‘patients demand treatment despite lack of evidence for effectiveness’ [11]. A qualitative study using direct observation of GPs in two UK practices found GPs rarely searched for, appraised and applied evidence directly from research. Instead, they relied upon what the investigators termed ‘mindlines.’ These were internalised tacit practice guidelines, derived from knowledge drawn from their own reading, training and practical experience, and from social interaction among practice colleagues, specialists, pharmaceutical company representatives and patients [46]. Whether seeking advice from a colleague, or reading clinically oriented material such as practice guidelines, several studies found the decision to act on that information depended on trust, rather than any attempt to analyse the information critically [30, 33, 35, 47]. A focus group of 5 to 7 GPs in the UK explored factors influencing uptake of new evidence. They identified a tendency to look for evidence that fitted pre-existing beliefs [33]. They also identified a belief among GPs that they should be conservative in the implementation of new evidence, as part of their role in ensuring the patient’s safety [33].

### Use of pre-appraised sources of evidence

A preference emerged among GPs for acquiring clinical information passively, through the post or electronically (sometimes described as information ‘push’ [48]) rather than engaging actively in their own independent search (described as information ‘pull’ [48]). It was a strong theme, emerging from 10 qualitative studies. [11, 26, 27, 33, 34, 36, 40, 42, 46, 49]. *‘I think EBM is predicated upon there being well-supported and financed independent reviewers who are doing the meta-analyses and the broader views which have become the key to evidence-based medicine and then selecting which ones are quality enough to include. It's just not practical for the family practitioner to be able to do that, even if you do have the tools, the time just isn't going to be there’* [27]. Surveys showed a stronger preference amongst GPs for using pre-appraised evidence than for seeking evidence themselves, [9, 10] and also showed pre-appraised evidence was perceived as more appropriate to general practice [9–11]. Surveys conducted in the period between 1997 and 2009 showed a minority of GPs had used MEDLINE [9, 13, 50] and the Cochrane Library [9, 13]. GPs are more inclined to rely on personal experience, [38] or other opinion-based sources of information [12] than to use these databases. One survey found the resources endorsed by the greatest proportion of GPs as ‘very useful’ were evidence-based guidelines (55%) and journals containing evidence summaries (52%); access to MEDLINE, and primary research articles were both rated ‘very useful’ by fewer GPs (27% and 25% respectively) [11]. Requests for improved formats of evidence [12, 14] may suggest a preference for evidence that is delivered to the GP, pre-appraised and ready to apply where appropriate. In a survey of 104 GPs in Australia, the suggestion was proposed that even guidelines could be summarised and collated to facilitate EBM [14].

### Integration of best evidence with clinical expertise, and the needs of the individual patient

There is a belief that evidence must be integrated with experience, and tailored for individual patients. This was among the strongest themes to emerge from qualitative studies, with numerous expressions of the principle that evidence should be integrated with both knowledge gained from experience, and considerations relating to the individual patient. [8, 26–29, 31–33, 35–37, 39–42, 45, 47]. Evidence on its own is perceived to be inadequate: *‘The guidelines will only take you so far and they're a useful stepping off point, but each individual case has so many factors at play beyond what the guidelines cover and that's where the art and the pleasure of medicine comes in using your clinical judgement to realize that what the protocol says doesn't apply to that person or it applies in a different way. That takes a good understanding of what the meaning is behind the protocol so I don't think you're ever just a technician’* [27]. In one UK survey, from a random sample of 500 GPs, 90% of 302 respondents claimed to integrate evidence and clinical experience [15]. The study was vulnerable however, to both responder bias (giving the response perceived to be desirable) and non-responder bias (non-responders potentially less likely than responders to integrate evidence with experience). A survey of 116 family practitioners in Turkey found 103 (89%) disagreed with the statement that EBM devalues clinical experience. While this finding does not provide direct support for the notion that both EBM and clinical experience should be integrated, it does at least suggest that the two entities were not widely considered to be mutually exclusive. GPs do appear to acknowledge the importance of personal clinical experience in making decisions [12, 38]. Finally, in a survey among 107 Australian GPs, in response to the prompt: ‘Is there anything that you believe would assist you generally in making clinical decisions about the care of your patients?’ 10 (9%) suggested mechanisms for tailoring evidence to the individual patient, and 5 (5%) suggested mechanisms for informing patients and eliciting their preferences [12]. This principle has not percolated fully through general practice however. A variable proportion of GPs actually regard patient expectation as a *barrier* to EBM, rather than a necessary component [9, 10, 12–14].

### Communication skills

There is a perceived need for competence in explaining complex information, including the risk and benefit of proposed interventions*.* This theme arose within a single qualitative study, [42] in which GPs offered perspectives on the ingredients of a successful ‘evidence-based patient choice’ consultation. GPs expressed the view that risks and benefits should be described for each treatment option, including that of no treatment [42]. In a survey conducted among Australian GPs, 20/60 (33%) agreed that insufficient skill to explain the implications of research to patients is a barrier to EBM [11]. Other surveys, however, have demonstrated that the level of understanding among GPs, of technical terms relating to expression of risk and benefit, is low [9–11, 13]. GPs value an ability to establish a doctor-patient relationship that is conducive to implementation of evidence. An emergent theme was that successful implementation of such evidence can only be achieved after the patient’s trust has been won, and that this requires empathy and the establishment of rapport: *‘So you need to get a rapport, and the trust of the patient, there’s no good enforcing treatment’* [45]. *‘We have to know, aside from diagnosing the patient, what he or she feels, and empathise with the person’* [28]. The ingredients of a successful ‘evidence-based patient choice’ consultation included elements normally expected of any doctor-patient encounter: trust, respect, honesty and partnership building [42]. Support for this theme was offered by the survey of 107 Australian GPs, in which 102 (95%) preferred some degree of patient involvement in decision-making [12]. The GP is required to educate patients about the nature of their condition, and also to guide them towards other relevant sources of information [42]. GPs participating in focus groups conducted in Belgium viewed patients as ‘consumers’ of health care, who could be taught to approach health information critically, though some envisaged a role here for consumer organisations [26]. The survey finding from 60 Australian GPs that 20 (33%) perceived a lack of skill in explaining the implications of research to patients as a barrier to EBM [11] suggests that an ability to educate patients would be valued by some GPs.

### Skill in searching for evidence

Some GPs believe that they should be able to conduct searches for research evidence. This emerged as a theme from four qualitative studies. [11, 26, 30, 49] GPs participating in a workshop exploring the management of information concluded that EBM skills, particularly those needed to search for evidence, should be included in medical curricula [49]. The perceived need for search skills emerged in more equivocal terms in interviews among 50 GPs in Australia, in which 21 (42%) viewed literature searching as a useful skill ‘in theory,’ but also perceived that time constraints would prevent implementation in practice [11]. Focus groups exploring EBM implementation found that most participating GPs were willing to search for evidence with the patient during a consultation [26]. A lack of search skills was found to be an obstacle to EBM when GPs were observed searching for evidence to answer clinical questions, [30] but this study did not directly address the question of whether literature searching is a necessary part of the GP’s skill set. Surveys indicated that awareness of major literature search databases such as MEDLINE [9, 13] and the Cochrane Library [9, 10, 13, 44] were variable, and that few GPs have been trained in conducting searches [9, 10]. Other survey findings also challenged the credibility of this theme: a variable proportion of GPs regarded lack of search skills as a barrier to EBM; [11, 12, 14, 16]. GPs preferred pre-appraised evidence to searching; [9, 10] and thought use of pre-appraised evidence to be more appropriate to general practice [9–11].

### Skill in critical appraisal of evidence

Some GPs perceive a need for competence in critical appraisal of research evidence. However, this theme arose from only one qualitative study, conducted among GPs practicing in Australia: three quarters of the 50 GPs interviewed agreed that GPs need some ability to critically appraise medical literature [11]. One survey supported this theme directly: conducted amongst 116 family practitioners in Turkey, it found that nearly all, 114 (98%), agreed that physicians must critically appraise literature [44]. Two surveys indicated widespread self-perceived ability to critically appraise research literature [15, 44]. One survey explored the frequency at which GPs critically appraise literature: conducted among 302 GPs in UK, it found that 37% claimed to critically appraise literature at least ‘sometimes.’ Evidence gleaned from other surveys was less supportive. One survey conducted in Jordan [13] explored 141 GPs’ views regarding the most appropriate method of EBM in general practice, between learning and using EBM skills, finding/applying evidence summaries, or using guidelines and protocols. No significant preferences were found. Another three surveys exploring the same question found a clear preference for guidelines over learning and using search or critical appraisal skills [9–11]. Furthermore, three surveys found only a small proportion of GPs (range 7% to 23%) agreed that a lack of skill in critical appraisal is a barrier to EBM [10, 12, 13]. One further survey among GPs who had participated previously in research (and therefore potentially vulnerable to selection bias) found a higher level of agreement at 55% [11]. As previously noted, GPs prefer to use pre-appraised sources of evidence rather than seek and critically appraise evidence themselves [9, 10]. Three surveys reported widely variable proportions of GPs claiming to have received training in critical appraisal [9, 10, 44]. GPs’ have a poor understanding of technical terms that have traditionally been key elements of critical appraisal teaching [9–11, 13]. This poor understanding was concordant across four surveys, comprising 638 GPs, in 3 countries and spanning 11 years between earliest and latest publication dates.

### ‘Straw model’ competency framework derived from the systematic review

After mapping the seven themes derived from the systematic review to the core principles of adult learning theory, [25] the following ‘straw model’ competency framework was constructed for experts to work on in the Delphi process:

#### Competency 1

The EBM competent GP reflects on instances in which risk and benefit were difficult to explain to a patient, and develops a concise, clear explanation for future use.

#### Competency 2

The EBM competent GP demonstrates an ability to identify decisions they found challenging, and to reflect upon their reasoning in terms of ‘4Es’: Evidence (availability, quality and applicability), Experience (their own accumulated on-the-job knowledge), Empathy (appropriate consideration of the patient’s perspective, capacity and circumstances) and Environment (the influence of external factors such as time pressure or access to resources).

#### Competency 3

The EBM competent GP demonstrates a commitment to checking newly acquired knowledge from colleagues, media, pharmaceutical companies and other sources against the best available evidence, using secondary sources such as guidelines, evidence-based summaries and systematic reviews.

#### Competency 4

The EBM competent GP demonstrates a commitment to efficiently addressing clinical uncertainty, using secondary sources of evidence, and the best available information technology.

#### Competency 5

The EBM competent GP works with colleagues to share the tasks of identifying and prioritising clinical questions, finding, appraising and presenting evidence, and refreshing knowledge of EBM concepts and resources.

#### Competency 6

The EBM competent GP demonstrates advocacy and leadership in identifying and addressing barriers to EBM within their practice.

### Themes derived from the Delphi process

The variance for mean overall ratings between Round 1 and 2 of the Delphi process reduced for five out of six competencies, suggesting a degree of consensus. There was little concordance of rankings between panelists, beyond that which would be expected by random chance, as indicated by a Kendall’s W statistic of 0.132. Thematic analysis resulted in seven themes, representing an integration of evidence from the literature, with evidence from a consensus of opinions from experts in general practice and EBM:

1. Decisions are influenced by information drawn from a complex array of sources.
2. There are social, cognitive, informational and organisational barriers to the notion of ideal EBM.
3. EBM involves tailoring decisions to the needs and perspective of the individual patient.
4. A sense of pragmatism must underpin the approach to seeking and critically appraising evidence, and to the development of competence in EBM, given the constraints implicit in general practice.
5. EBM requires commitment.
6. Reflection is a continuous background process, and should be objective.
7. EBM is difficult to demonstrate using currently available performance appraisal tools.

### Final competency framework

Among the themes derived from the mixed-methods systematic review, two were considered to lack credibility – (1) skill in searching for primary sources of best evidence, and (2) skill in critical appraisal. In each case, survey findings failed to support the enthusiasm expressed for these attributes in qualitative studies. The remaining five themes were compared with those derived from the Delphi process. Much meaning was shared between the two sets of themes, and they were condensed into a simpler final framework of three competencies:

1. Mindfulness in one’s approach towards EBM itself, and to the influences on decision-making.
2. Pragmatism in one’s approach to finding and evaluating evidence.
3. Knowledge of the patient, as the most useful resource in effective communication of evidence.

## Discussion

A systematic review provided a literature-based, thematic exploration of what EBM means in general practice; a Delphi process developed those themes further in light of the consensus opinion of experts in EBM and general practice. Integration of both sets of data resulted in a simple real-world competency framework with potential application in training, and in the individual pursuit of continuing professional development, and which could guide routine practice: (1) *mindfulness* in one’s approach towards EBM, and the influences on decision-making; (2) *pragmatism* in one’s approach to finding and evaluating evidence; and (3) application of one’s *knowledge of the patient* in communication of evidence. Mindfulness originated in Buddhist practice and is described as the process of developing an open and unbroken awareness of present-moment cognitive-effective and sensory experience [51]. It has been demonstrated in a randomised controlled trial to reduce and prevent burnout, promote positive attitudes among health professionals, strengthen patient-provider relationships, and enhance well-being among practitioners [52]. It has generated considerable interest in medical education, with many medical and dental schools offering courses [53]. We are not the first to propose mindfulness as an approach to EBM. Epstein presented mindfulness as a link between relationship-centred care and Evidence-Based Medicine [54]. His proposition that ‘all data, regardless of their completeness or accuracy, are interpreted by the clinician to make sense of them and apply them to clinical practice’ [54] reflects our systematic review findings. We regard mindfulness as a non-judgemental, global awareness of the physical and mental processes at play in the present moment. In the context of our competency framework, it means maintaining an open attitude towards EBM, aware of its strengths and limitations. It refers also to an awareness of the various subjective notions, biases and entrenched beliefs – among all parties - that might influence decisions. It embraces their influence as an inevitable part of the cognitive process of EBM, and asks only that they be acknowledged. Mindful practice allows space for unconscious incompetence to rise to the surface and be identified [54]. It entails being sympathetic towards one’s self, recognising the constraints imposed by the working environment, and the factors influencing the patient’s perspective. ‘Pragmatism’ in this context refers to a realistic ‘just-in-time’ approach to finding useful, valid evidence amidst the turmoil of a typical working day. Accepting that it is not feasible to conduct systematic searches and critical appraisals within or between consultations, it means becoming familiar with, and adept at using trusted online sources of pre-appraised evidence. ‘Knowledge of the patient’ refers to the particular ability of the general practitioner to ‘read’ their patients – to know instinctively when it is appropriate to communicate evidence, and when it is best saved for another opportunity. This is enhanced by the cultivation of a solid, therapeutic doctor-patient relationship.

There are similarities between our real-world framework and the other EBM competency frameworks and curricula we have reviewed. Mindfulness, and knowledge of the patient as a resource for evidence communication are both echoed in several instances (Table 3).

**Table 3:** Examples of convergence between the real-world competency framework, and other frameworks/curricula

Real-world competency	^a^Other frameworks/curricula
Mindfulness in one’s approach towards EBM itself, and to the influences on decision-making.	Be aware that beliefs and values, in doctor and patient, influence the interpretation of research results in support of potentially divergent views’ (RACGP, CTRT 1.3).
	Be aware of your own knowledge, limitations, biases and values that influence the way one practices medicine (RACGP, CTRT 4.4).
	Be aware of external influences on your own practice (e.g. pharmaceutical companies, media) and be confident in dealing appropriately with these influences’ (RACGP, CTRT 4.5).
	Ensure you understand the evidence or experience underpinning your own understanding, and be clear when you are stating an opinion based on experience rather than evidence’ (RCGP, EF0.3).
	Acquire the research and academic skills required of a GP that aid decision-making, which include a non-judgemental evidence-based approach to problem solving and recognising how individual bias may affect your interpretation’ (RCGP, 3.1).
	Take into account psycho-social factors, learning disabilities, the vulnerability of patients, and cultural backgrounds when taking an evidence-based approach and apply the findings on both an individual and a population level’ (RCGP, 3.5).
Knowledge of the patient, as the most useful resource in effective communication of evidence.	Communicate the evidence for management, diagnosis or screening to patients in a manner that is both understandable to the patient, and is patient centred (RACGP, CTRT 1.1).
	Being aware of how you impart information about evidence so that patients can best understand relevant evidence and be helped in making a decision (RCGP, EF0.1).

^a^ *RACGP* Royal Australian College of General Practitioners, *RCGP* Royal College of General Practitioners (UK)

All the frameworks and curricula have noticeably moved away from the stepwise, sequential nature of the traditional model, perhaps recognising the difficulty of integrating such a process with routine work. There are however, fundamental differences. The other frameworks cleave more closely to the competencies of the traditional EBM model. Our systematic review findings do not support this approach. We found consistent evidence that the traditional model has failed to gain traction in general practice: direct observation of GPs has revealed no engagement in the traditional five steps of EBM [46]. Even self-reported EBM activity, which might be susceptible to response bias, is disappointingly low: in a survey among 302 UK GPs, 39% claimed to identify gaps in knowledge frequently; 28% to frequently track down evidence; and only 8% to frequently appraise literature critically [15]. Notably, the requirements for search and appraisal skills were the only themes to have lacked credibility in our systematic review. Our findings challenge the assumption that they can be incorporated into the working day. Some qualification of our approach is required here however: we recognise that search and appraisal skills are a vital part of the medical curriculum, and should be maintained throughout a GPs’ career. Indeed, some knowledge of search and appraisal is necessary to make judgements about the quality of pre-appraised evidence. We also acknowledge that occasionally, a clinical question will only be addressed by searching and critical appraisal of primary research. But we cannot pretend that it is feasible to expect GPs to search MEDLINE, and conduct sound critical appraisals of primary research during routine work.

It is clear from our data that the traditional model of EBM has encountered considerable challenges in general practice. The real-world approach we propose here offers two advantages: simplicity, and flexibility. We propose that a simpler, more streamlined framework of only three competencies might be easier to integrate into routine work. It is more flexible in that it allows some room for manoeuvre in the imperfect, real world. It assumes the position that EBM is about individual GPs and individual patients making the best possible decisions in the prevailing circumstances. To illustrate how a GP might demonstrate the real-world competencies in practice, we describe a clinical scenario.

*Fiona’s last patient on Friday afternoon is Reg, a 70-year-old retired army officer. He’s well turned out, having put on a tie to see the doctor. He tells Fiona he hasn’t been sleeping in the last six weeks since his wife died. Fiona’s colleague had prescribed a sleeping tablet, and that had helped. He moves on quickly in response to Fiona’s enquiries about his mood, and says all he needs is some more of those sleeping tablets. Fiona recalls a complaint made by Reg some years ago, when she suggested a referral for cognitive behavioural therapy (CBT) for a bout of anxiety. She’s about to type ‘Temazepam 10 mg’ when she remembers insomnia had been discussed at a Practice Based Small Group Learning (PBSGL) session she attended a few weeks ago. She considers whether she might have been a little rash with her decision to prescribe. Perhaps it was because it’s Friday afternoon and she’s tired – or maybe because she didn’t want to upset Reg again* (**mindfulness**). *She asks Reg if he would mind waiting a second while she looks for some useful information on the computer. She opens the TRIP database (* *www.tripdatabase.com* *) she had used in the PBSGL meeting* (**pragmatism**)**.** *She types ‘insomnia’ in the search box, and opens the first item listed, which is an evidence-based synopsis from BMJ Clinical Evidence (* *www.clinicalevidence.bmj.com* *). Casting her eyes quickly down the page, she reads that ‘the evidence for effectiveness of CBT for insomnia is convincing, whereas the evidence for exercise programmes and timed exposure to bright light is less clear. She reasons that a suggestion of exercise might however be more acceptable to Reg than a course of CBT or bereavement counseling* (**knowledge of the patient**)*. She prints off an information sheet on insomnia from* *www.patient.co.uk* *and uses a hypnogram to explain sleep cycles. She also explains how sleeping tablets only work at the start of sleep, and that they can soon produce dependence. She explores the various options with Reg, broaching CBT very tentatively* (**knowledge of the patient**). *She’s not surprised when he seems more inclined to try a programme of exercise, and she manages to secure his agreement to try that, and report back in a week. After Reg leaves, she wonders whether 20 min was a bit long for such a consultation* (**mindfulness**). *She decides to print off a batch of information sheets and a hard copy of the evidence synopsis to keep in her desk drawer.*

To our knowledge, this methodological approach to the development of a competency framework is novel. We were unable to identify a clear consensus regarding the best approach within the field of medical education. Myriad techniques have been used, including practice profiling, task analysis, [55] focus groups, questionnaires, [56] critical incident interviews [57] and direct observation [57] to name but a few. Systematic review has been used previously to identify competencies [58, 59]. We chose this approach because it offered an efficient way of tapping a vast pool of useful data. Other researchers have used the Delphi process [60–63]. It is an efficient way of generating meaningful data from experts in the field. It does this without allowing any individual voice to dominate; and in contrast to other approaches, such as critical incident technique, it does not focus exclusively on conspicuous elements of the role [64]. Our use of systematic review together with the Delphi process allowed us to triangulate a large amount of data from qualitative and quantitative research literature, with the opinions of experts in both general practice and EBM. To our knowledge, this is the most comprehensive exploration of EBM competence in general practice. Further research is needed however, to validate the competency framework as an educational and on-the-job tool. This might involve seeking expert consensus, focus group work, or randomised controlled trial. A validated framework could have a number of useful applications that could promote and consolidate EBM in the general practice setting: it could be incorporated into training curricula, each competency accompanied by vignettes illustrating how it could be demonstrated in a variety of contexts; it could contribute to GP job interview processes; and it could be presented as a package for GPs to include in the personal development component of the performance appraisal process. The competencies (with illustrative vignettes) could be included as a standard component of appraisal tools. This would address the theme arising from the Delphi process, that EBM is difficult to demonstrate using currently available performance appraisal tools. Furthermore, appraisers could be trained to spot when the competencies are being met, or arise as a learning need.

### Limitations

There were a number of limitations to the mixed-methods systematic review. The search strategy included studies that addressed engagement with EBM as an overarching approach to patient care, and excluded those that addressed individual steps in the traditional model of EBM as published in the Sicily Statement [3]. It was felt that isolated steps did not in themselves constitute the overall EBM approach to patient care. Studies that evaluated educational interventions were excluded because initial scoping of the literature revealed that the traditional steps of EBM were often assumed a priori to be the desired competency outcomes. Furthermore, trials in education are notoriously prone to contamination (participants receiving the teaching intended for those in another group) [65] and lack of external validity (where few situations mirror the conditions or participants in the original study, making conclusions inapplicable elsewhere) [66]. We also excluded studies in languages other than English. This was because resources for translation were unavailable. It is possible that some relevant studies have been missed as a result, though most relevant general practice research is likely to be in English given its prominence as a specialty in Anglophone countries. As a result of logistic constraints, only one reviewer made decisions on selection of relevant studies, assessment of their quality and extraction of data. Qualitative data were also coded by a single reviewer. This may impair the reliability of findings, and the review would have been strengthened by provision of a second independent reviewer to perform the same functions, blinded to the decisions made by the first reviewer. The quality of included studies was limited. There is no validated framework available to date, for the assessment of quality in observational studies. This review, like others [19, 67] used the recommendations of the STROBE initiative [20]. This was designed as a checklist for authors submitting studies for publication to peer-review journals to ensure a minimum methodological standard, and was not intended for the use to which it has been applied in this and other reviews. In absence of an alternative, validated framework, it provided a pragmatic and explicit alternative, which users of this review can easily critique.

The Delphi process was also subject to limitations. Because of the limited availability of experts willing to give precious time to a relatively time-consuming process, the size of the expert panel was at the lower end of those reported as acceptable [68]. A small panel could arguably result in under- or over-representation of certain types of expertise. However, a thorough and systematic approach was taken to finding suitable candidates, based on criteria determined a priori. These criteria were themselves based on suitable experience in general practice, and one or other of either academic experience in EBM, or full-time work as a GP. Among the academic GPs, panelists were invited on the basis of their published research interests, which were deliberately aligned with the themes emerging from the systematic review. It might be argued that a further potential limitation arises from the self-recruitment of the researcher as a member of the expert panel. Anonymity, however, and the involvement of a decision committee at each stage provided protection from over-representation of the researcher’s opinion. Coding by a single investigator was not ideal, but the threat to reliability was mitigated by repeat coding after a number of days, and the finding that the coding did not change over this time. This consistency of coding was not surprising, given that qualitative data were largely arranged around very specific, well-defined competencies. There was little concordance of rankings between panelists, beyond that which would be expected by random chance, as indicated by a Kendall’s W statistic of 0.132. Some reassurance can be taken however, from the reduction of variance for mean overall ratings between Round 1 and 2, for five out of six competencies, which suggests a degree of consensus. The investigation also has strength, arising from full engagement without attrition, by a panel representing a wealth of expertise. Also, the anonymity implicit in the Delphi process ensured that no expert voice was dominant, and all panelists would have felt unfettered in their responses. Indeed, an abundance of valuable qualitative data were generated from free-text comments.

## Conclusion

Current curricula and competencies for EBM do not acknowledge the constraints and competing demands of real-world general practice. Given low levels of engagement with EBM in general practice, future development and refinements should take into account the available evidence from empirical research regarding the knowledge, skills, attitudes and behaviour of GPs in this context. We synthesised this evidence, and proposed an EBM competency framework for real-world general practice. We have illustrated how this approach might be applied in practice, and considered its potential as a learning tool. It will require further validation as an educational and on-the-job resource.

## Additional file

Additional file 1: Characteristics of included studies. (PDF 86 kb)

## Abbreviations

CBT: Cognitive behavioural therapy
EBM: Evidence-based medicine
PBSGL: Practice based small group learning
RACGP: Royal Australian College of General Practitioners
RCGP: Royal College of General Practitioners
STROBE: Strengthening the reporting of observational studies in epidemiology
