# Supplementary material for: A real-world approach to Evidence-Based Medicine in general practice: a competency framework derived from a systematic review and Delphi process
Source: BMC Med Educ. 2017 May 3;17:78. doi: 10.1186/s12909-017-0916-1 (PMC5415750; doi:10.1186/s12909-017-0916-1)
Supplement: Additional file 1: — Characteristics of included studies. (PDF 86 kb) [file 12909_2017_916_MOESM1_ESM.pdf]

Table 1a. Characteristics of included studies: cross-sectional surveys (N=14)

	Design	Subjects	No. GPs invited who fit inclusion criteria*	GP response rate§	Competency-related outcomes explored
**Barghouti F, 2009 Jordan[13]**	Self-administered questionnaire	Family physicians	200	70%	EBP knowledge, understanding, attitudes and awareness; perceived barriers to EBP.
**Callen J, 2006 Australia[9]**	Self-administered questionnaire	GP members of a Division of Medical Practice	434	31%	EBP attitudes, awareness, understanding and implementation; perceived barriers to EBP.
**Hannan A, 1998 UK[69]**	Self-administered questionnaire	GPs, surgeons and school teachers	17	47%	Use of research compared between professional groups.
**Kahveci R, 2009 Turkey[44]**	Self-administered questionnaire and interview-based questionnaire	GPs, family medicine trainees and family medicine specialists	770	31%	EBP knowledge, attitudes and opinions on future practice; EBP training; perceived barriers to implementation.
**McColl A, 1998 UK[10]**	Self-administered questionnaire	GPs	450	67%	Attitude to EBP; ability to access and interpret evidence; perceived barriers to EBP; opinions regarding best method of moving from opinion based to evidence based medicine.
**McKenna H, 2004 UK[16]**	Self-administered questionnaire	GPs and community nurses	356	57%	Perceived barriers to EBP.
**Robinson G, 2000 UK[70]**	Self-administered questionnaire	GPs	295	84%	Attitudes regarding the importance of primary care research; interest in and use of research; interest in, and experience of doing research.
**Salisbury S, 1998 UK[71]**	Self-administered questionnaire	GPs	184	98%	Relationship between GP characteristics and implementation of evidence-based prescribing initiatives.
**Samuel O, 1997 UK[50]**	Self-administered questionnaire	GPs and GP trainers	1739	28%	Rates of EBP activities in the prior 2 weeks, and of desire for training in use of Medline; opinions regarding EBP.
**Siriwardena A, 2007 UK[72]**	Self-administered questionnaire and written knowledge test	GPs and secondary care physicians	NR	NR	EBP knowledge and attitudes.
**Taylor J, 2002 Australia[14]**	Interview-based questionnaire	Rural and remote-practicing GPs	104	86%	EBP attitudes and self-reported implementation; perceived barriers to EBP and opinions regarding potential solutions.
**Tracy C, 2003 Canada[73]**	Self-administered questionnaire	Family physicians (members of Canadian College of Family Physicians)	1037	42%	Relationships between physician attitudes toward EBM, contextual factors, and decision-making; factors that contribute to clinical decisions that contradict best evidence.
**Trevena L, 2007 Australia[12]**	Open-ended, telephone survey	GPs	155	69%	Information sources for clinical decisions; barriers to EBP; suggested strategies to improve decision-making; preferences regarding patient involvement.
**Upton D, 2006 UK[15]**	Self-administered questionnaire	GPs and hospital doctors	500	60%	Self-rated EBP knowledge and skills; self-reported application of EBP; perceived barriers to EBP. Comparisons of outcomes between GPs and hospital doctors.

*General or family practitioners, trained or in training, and working in the community. § Response rate as defined in Last J.M.(Ed). A Dictionary of Epidemiology. 4th ed. USA: Oxford University Press; 2001. NR Not reported

Table 1b. Characteristics of included studies: qualitative studies (N=21)

	Design	Participants	No. GP participants	Competency-related outcomes explored
**Adams J, 2000 UK[29]**	In-depth interview	GPs practicing complimentary and alternative medicine (CAM)	25	EBP perceptions; relationship with CAM.
**Armstrong D, 2002 UK[45]**	Semi-structured interview	GPs from four health authorities	80	Management of depressed patients; reaction to the availability of new drugs.
**Calderón C, 2011 Spain[28]**	Focus groups	Family practitioners working in 47 public health centres	67	EBP perceptions in primary care context.
**Ely J, 2002 US[30]**	Participant observation	Academic generalist doctors, family doctors and medical librarians	23	Obstacles encountered while searching for evidence based answers to doctors' questions.
**Ford S, 2002 UK[31]**	Semi-structured interview	GPs, hospital doctors, practice nurses, academics and lay people	11	Barriers to implementation of the 'evidence-based patient choice' consultation.
**Ford S, 2003 UK[42]**	Semi-structured interview	GPs, hospital doctors, practice nurses, academics and lay public	11	Elements and skills required for a successful 'evidence-based patient choice' consultation.
**Freeman A, 2001 UK[32]**	Balint-style groups	GPs	19	Perceived barriers to EBP.
**Gabbay J, 2004 UK[46]**	Semi-structured interview, non-participant observation and analysis of documents/materials	GPs, nurses, phlebotomist, and associated medical staff in one practice providing initial data; transferability of theoretical model checked with general practitioners in a second practice	NR	Use of evidence in individual and collective healthcare decisions; social and organisational processes by which evidence, information, and knowledge become knowledge in practice.
**Hall L, 1999 UK[33]**	Focus groups	GPs, consultants and hospital nurses	18	Attitudes to clinical effectiveness and behaviour change in the context of professional and social relationships within the current organisation of the National Health Service.
**Hannes K, 2005 Belgium[26]**	Focus groups	GPs (mix of academics, recruits from local GP peer groups, and a course in EBP)	31	Perceived barriers to EBP; strategies to overcome barriers.
**Lipman T, 2004 UK[74]**	Semi-structured interview	GPs active in research network or EBP workshops	11	Factors influencing decision-making regarding anticoagulation for atrial fibrillation.

Continued

Qualitative studies (continued)

**Lorenz K, 2005 US[47]**	Focus groups	Primary care providers (paediatricians, family practitioners, general internists and physician managers)	NR	Factors prompting search for evidence; search strategies and sources; comparison of clinician and physician manager approaches.
**Mayer J, 1999 Australia[35]**	Focus groups	GPs	27	Attitude to EBP; factors affecting consideration and use of evidence from a selection of guidelines within consultations.
**Mears R, 2000 UK[8]**	Semi-structured interview	GPs, based in research-based general practice or involved in continuing medical education	5	Factors influencing decision making.
**Putnam W, 2002 Canada[37]**	Focus groups	Family physicians	50	Perspectives on use of evidence, and it's influence on practice in the context of managing patients with cardiovascular disease.
**Short D, 2003 UK[39]**	Semi-structured interview	GPs	15	Factors that lead GPs not to prescribe aspirin in stroke patients, contrary to recommendations in guidelines.
**Skoglund I, 2007 Sweden[40]**	Focus groups	GPs	16	Thoughts about EBP and prescribing.
**Summerskill W, 2002 UK[41]**	Focus groups and semi-structured interviews	GPs	14	Factors influencing use of secondary prevention in management of patients with coronary heart disease.
**Tomlin Z, 1999 UK[75]**	Semi-structured interview	GPs	24	Definitions of effective health care; reasons for not practicing effectively according to respondents' own criteria; sources of information used to answer clinical questions; reasons for making changes in clinical practice.
**Tracy C, 2003 Canada[27]**	Semi-structured interview	Family physicians	15	EBP attitudes and experience; influence of patients' preferences on decision-making; role of intuition in family practice.
**Wood F, 1995 UK[49]**	In-depth, structured and semi-structured interviews, non-participant observation and workshop	GPs and other practice staff in two studies (Trent and Anglia/Oxford), reported together	62	Information-related behaviour of GPs; reasons for information needs; satisfaction with information seeking; sources of information; communication of information; effects of national health service reforms on information needs; opinions on future needs, and appropriate strategies; guidelines for best information practice.

NR not reported

Table 1c. Characteristics of included studies: mixed methods studies (N=3)

	Design	Participants	No. GPs invited who fit inclusion criteria*	Survey response rate (%) *	No. Participants in qualitative study	Competency-related outcomes explored
**Patterson J, 1999 UK[36]**	Mixed methods (Self-administered questionnaire and in-depth interview)	GPs	44	82%	14	Attitudes to EBP; comparison of attitudes in the context of patient care consultations, and in purchasing; change in attitudes over time; perceived barriers to EBP.
**Rohrbacher R, 2009 Germany[38]**	Mixed methods (interviews, semi-structured questionnaire)	GPs, specialist physicians and patients	NR	NR	104	Reasons underpinning treatment selection for five selecte medical conditions; attitude to EBP; patient satisfaction.
**Young J, 2001 Australia[11]**	Mixed methods (semi-structured interviews and SAQ)	GPs	60	100%	50	Current use of information technology; views about EBP; self-reported understanding of EBP terms; perceived barriers to EBP; preferred EBP resources; perceptions regarding EBP, barriers, EBP-supportive strategies and preferred sources of information when faced with clinical uncertainty.

NR not reported. *Response rate as defined in Last J.M.(Ed). A Dictionary of Epidemiology. 4th ed. USA: Oxford University Press; 2001
